# Single step calcium sulfate scale removal at high temperature using tetrapotassium ethylenediaminetetraacetate with potassium carbonate

**DOI:** 10.1038/s41598-022-14385-6

**Published:** 2022-06-16

**Authors:** Mobeen Murtaza, Sulaiman A. Alarifi, Mohammed Yousef Rasm, Muhammad Shahzad Kamal, Mohamed Mahmoud, Mohammed Al-Ajmi

**Affiliations:** 1 Center for Integrative Petroleum Research (CIPR), King Fahd University of Petroleum and Minerals, Dhahran, 31261 Saudi Arabia; 2 Department of Petroleum Engineering, King Fahd University of Petroleum and Minerals (KFUPM), Dhahran, 31261 Saudi Arabia; 3 Petroleum and Energy Logistics and Services Co. (Petrogistix), Al-Khobar, 34227 Saudi Arabia

**Keywords:** Energy science and technology, Fossil fuels

## Abstract

Calcium sulfate (CaSO_4_) scale has been identified as one of the most common scales contributing to several serious operating problems in oil and gas wells and water injectors. Removing this scale is considered an economically feasible process in most cases as it enhances the productivity of wells and prevents potential severe equipment damage. In this study, a single-step method utilizing potassium carbonate and tetrapotassium ethylenediaminetetraacetate (K4-EDTA) at high temperature (200 °F) has been used to remove CaSO_4_ scale. The CaSO_4_ scale was converted to calcium carbonate (CaCO_3_) and potassium sulfate (K_2_SO_4_) using a conversion agent, potassium carbonate (K_2_CO_3_), at a high temperature (200 °F) and under various pH conditions. Various parameters were investigated to obtain a dissolver composition at which the optimum dissolution efficiency is achieved including the effect of dissolver pH, soaking time, the concentration of K4-EDTA, the concentration of potassium carbonate (K_2_CO_3_), temperature impact and agitation effect. Fourier transform infrared, X-ray crystallography, ion chromatography, stability tests and corrosion tests were carried out to test the end product of the process and showcase the stability of the dissolver at high temperature conditions. A reaction product (K_2_SO_4_) was obtained in most of the tests with different quantities and was soluble in both water and HCl. It was observed that the dissolver solution was effective at low pH (7) and resulted in a negligible amount of reaction product with 3 wt% CaSO_4_ dissolution. The 10.5-pH dissolver was effective in most of the cases and provided highest dissolution efficiency. The reaction product has been characterized and showed it is not corrosive. Both 7-pH and 10.5-pH dissolvers showed high stability at high temperature and minimum corrosion rates. The single step dissolution process showed its effectiveness and could potentially save significant pumping time if implemented in operation.

## Introduction

Scale is a common term used in the oil and gas industry to describe solid deposits that grow over time, blocking and hindering fluid flow^1^. Scale formation is a critical operational issue in surface and subsurface oil and gas equipment as it could occur at all stages during oil and gas production. Scale deposits around the wellbore clog the porous formation medium, making the formation impervious to fluids. Scale causes blockage of wellbore perforations, pipelines and valves, leading to equipment wear, corrosion and flow restriction, reducing oil and gas output^2^. Also, it is a significant cause of formation damage in both injection and production wells^3,4^. Scale formation in oil and gas fields is a costly problem due mainly to causing lower oil and gas production and requiring frequent down-hole equipment replacement, re-perforation of productive intervals, re-drilling of plugged oil wells and other remedial workovers needed to avoid its consequences. Scales caused by calcium carbonate, calcium sulfate, iron sulfide, strontium sulfate and barium sulfate are the most common scales in the oil and gas industry^5^.

Scales are categorized according to their mechanism of removal. Due to the insoluble nature of chemically inert scales in other chemicals, mechanical means must be utilized to remove this type of deposit. Chemically reactive scales are classed according to their solubility in water, acid, or chemicals other than water or acid. Sodium chloride is an example of a water-soluble scale that does not recommend dissolved using acid. Acid soluble scales are the most common type of scale. Calcium carbonate, for example, is a soluble acid scale. Calcium carbonate can be removed using hydrochloric acid, acetic acid, formic acid or sulfamic acid. Other acid-soluble scales are iron carbonate, iron sulfide and iron oxide (Fe_2_O_3_). Iron scales can also be removed using HCl and a sequestering agent.

The major scale contributing to various serious operating difficulties in oil and gas wells and water injectors has been identified as calcium sulfate. Calcium sulfate hard scale deposits that are well impermeable can limit formation permeability, lowering well injectivity and productivity. Calcium sulfate precipitation in down-hole equipment, such as electrical submersible pumps, can have a negative impact on well performance. Due to the overloading caused by this precipitation, the pump fails, causing significant damage to its components. As a result, expensive workovers are necessary.

Calcium sulfate crystallizes in two distinct and stable forms. At low temperatures (i.e., Temperature < 98 °C), gypsum (calcium sulphate dihydrate, CaSO_4_·2H_2_O) is created, whereas anhydrite, CaSO_4_, is the main form at high temperatures^6^. Gypsum, the most prevalent calcium sulfate scale in oilfields, is extremely difficult to remove. This is mostly due to its poor solubility limits in water, which are approximately 2.36 kg in 1 m^3^ of water at 77 °F. At higher temperatures, calcium sulfate becomes increasingly insoluble in water, reaching a concentration of 1.69 kg in 1 m^3^ of water at 194 °F. Apart from temperature, additional parameters affecting calcium sulfate solubility include the solution pH value and pressure. Calcium sulfate, in general, is more soluble at low pH levels and high pressures^7,8^.

The primary reason for calcium sulfate scaling during these activities is the combination of two chemically incompatible fluids. For example, when injected seawater containing a high concentration of sulfate ions is mixed with formation water containing a high concentration of calcium ions, calcium sulfate precipitates after its solubility limit are surpassed. Similarly, calcium sulfate precipitation can occur when spent acid solutions are mixed incompatibly with over flushed fluids.

Sulfate scales are often encountered in seawater applications and are troublesome since they do not dissolve in acids. They do, however, dissolve in chelate solutions with a high pH^9^. There were different dissolvers applied for the dissolution of gypsum and anhydrite scale. Riad et al. used soda ash as gypsum dissolver in 8″ Production Line in Gemsa Field in Egypt. The converted product was washed by post flushing with 15 wt% HCl^10^. Numerous hydroxides such as calcium hydroxide have been employed to remove gypsum deposits with varying degrees of success. The hydroxide dissolves the gypsum and leaves behind calcium hydroxide, which is soluble in acid. Although some success has been achieved with the hydroxides, their effectiveness has decreased in some circumstances due to a buildup of calcium hydroxide sludge within the system and the hydroxide coating created on the surface of the gypsum deposit. Additionally, as previously stated, the hydroxide technique requires an acid to wash away the reaction precipitate, which is normally undesired. Lawson used hydroxamic acid for the dissolution and disintegration of the gypsum^11^. Further, Calcium sulphate dihydrate (gypsum) scale has been prevented at temperatures ranging from 104 to 194 °F using organic phosphate ester as a scale inhibitor^12^. Wang et al. introduced a novel environmentally friendly type of scale inhibitor acrylic acid–oxalic acid–allypolyethoxy carboxylate (AA-APEM9) for the inhibition of the calcium sulfate scale^13^.

Scale removal can be performed without using organic or inorganic acids by applying chelating agents. Acids are toxic and corrosive to well tubing and downhole equipment, on other hand, chelating agents are more environmentally friendly, biodegradable and have a low corrosion rate. As chelating agents have a mild effect on sensitive downhole equipment such as electrical submersible pumps, they are recommended for eliminating inorganic scales. Two chelating agents, Ethylenediaminetetraacetic acid (EDTA) and diethylenetriaminepentaacetic acid (DTPA), have been extensively investigated in the treatment of scale^14,15^. Rhudy discussed the removal of calcium, barium and strontium scales from reservoir cores using EDTA and DTPA formulations^16^. According to Lakatos et al., DTPA and HEDTA were effective for dissolving barium and strontium sulfate scale^17^. One of the early applications of EDTA in the oil field was to remove calcium carbonate scale from the sandstone^18^. EDTA was also employed to clean brine heater tubes and boilers of calcium sulfate scale^19^. Additionally, EDTA was employed to clean clay assemblages of sulfate and carbonate mineral scale^20^. Tetrasodium glutamate diacetate (GLDA) was employed to remove gypsum, and it was demonstrated that the ammonium salt outperformed the sodium salt. Athey et al. demonstrated many uses of Hydroxyethyliminodiacetic acid (HEIDA) in scale removal processes^21^. It was demonstrated that HEIDA can store more calcium than Nitrilotriacetic acid (NTA) and was successful at dissolving calcium sulfate and barium sulfate scale when used in conjunction with a carbonate 'conversion' agent^22^.

Scale inhibitors are often the most cost-effective technique for calcium sulfate reduction^23^. Calcium sulfate precipitation requires, however, the use of a cost-effective treatment. Calcium sulfate scaling near-wellbore can be removed utilizing chemical dissolvers rather than mechanical methods. The usage of amino carboxylic acid salts as a chemical method has been intensively studied. The calcium sulfate dissolution is effective in EDTA compared to other acids. The calcium sulfate dissolves in live acids but later, it reprecipitates and causes severe formation damage. Hydrochloric acid is not a good solvent for CaSO_4_. The maximum solubility of calcium sulfate in HCl is only 1.8 wt% at 77 °F and atmospheric pressure. EDTA can dissolve gypsum and anhydrite in amounts of 43 and 34 g/L, respectively^19^. The calcium sulfate scale does not reprecipitate in EDTA dissolver. Cikes et al. used 8% tetrasodium EDTA to remove calcium sulfate damage in high-temperature gas-condensate well^24^.

CaSO_4_ scale dissolution was investigated recently by Murtaza et al. in which 5 wt% calcium sulfate and 8 wt% of potassium carbonate were allowed to react using water as a solvent at ambient condition for several hours. The pH of the reaction solution was 11.6. The reaction took place under continuous stirring using a magnetic stirrer. The reaction product was filtered and dried overnight to remove all the water. The solubility tests were performed using acids such as HCl and acetic acid. The solubility of reactant (calcium sulfate) and converted product (calcium carbonate) was evaluated by dissolving a fixed amount of solid in 10 ml of acid. The removal efficiency was calculated by the ratio of dissolved solid in acid to the original amount of solid added to the acid. For their method, due to the calcium sulfate scale not dissolving in acids such as acetic acid and HCl, a conversion method was applied where the calcium sulfate scale was converted to calcium carbonate by reacting calcium sulfate with potassium carbonate in high pH conditions. The resulting product is soluble in most of the acids such as acetic acid and HCl. This conversion was achieved at ambient conditions^25^.

**Figure 1:**
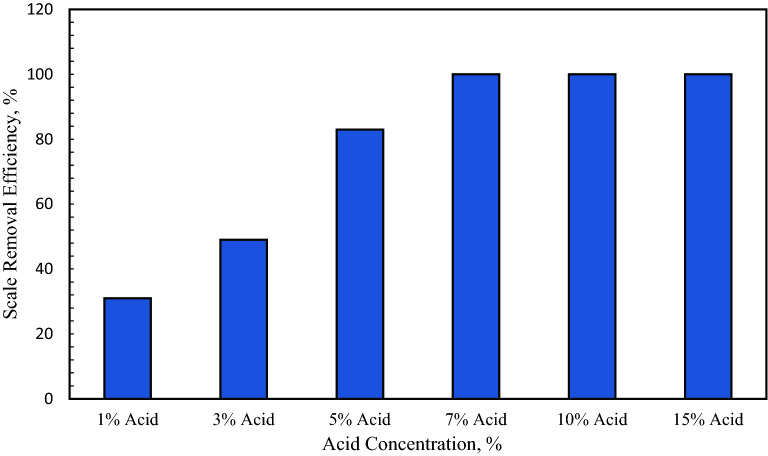
Effect of acetic acid concentration on calcium sulfate scale removal using the potassium carbonate conversion method^25^.

**Figure 2:**
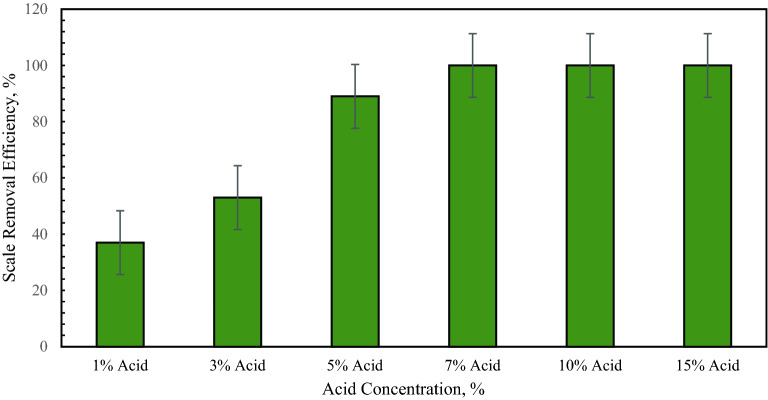
Effect of HCl acid concentration on calcium sulfate scale removal using the potassium carbonate conversion method^25^.

Figure 1 shows the percentage of calcium scale removal using the proposed conversion and by dissolving the converted product in acetic acid. For acetic acid, 1 g of the converted product was dissolved in acid. Using 1 wt% and 3 wt% acetic concentration, the scale removal efficiency was 31% and 49%, respectively. When the acid concentration was 5 wt%, the removal efficiency of 83% was achieved. The optimum concentration was 7 wt% for acetic acid, where all the converted product dissolved in acetic acid. A similar dissolution was noted at 10 wt% and 15 wt% acetic concentration^25^. Figure 2 shows the effect of HCl concentration on the removal efficiency of calcium sulfate scale using the conversion method. Like acetic acid, HCl acid also showed optimum performance at 7 wt% concentration, where 100% removal efficiency was achieved.

There are several studies that investigated using HCl and EDTA solutions for the dissolution of CaSO_4_ scale. Many of these studies were not conducted in detail and only studied limited parameters. In this study, we investigated the dissolution thoroughly under numerous parameters. The calcium sulfate scale dissolution was investigated by converting it to acid soluble product using a conversion agent, potassium carbonate (K_2_CO_3_), and tetrapotassium (K4) EDTA acid as a chelating agent at elevated temperature (200 °F). The high temperature is used to simulate the reservoir or bottomhole conditions when using this dissolver in operation. The conversion and dissolution were carried out in a single step, subjecting the calcium sulfate to a mixer containing K4-EDTA and K_2_CO_3_. Different parameters were varied to investigate the dissolution efficiency, including the effect of dissolver pH, soaking time, the concentration of K4-EDTA, the concentration of K_2_CO_3_, temperature impact and agitation effect. Fourier transform infrared (FTIR) and X-ray crystallography (XRD) characterization, stability tests and corrosion tests were carried out to test the end product and showcase the stability of the dissolver at high temperature conditions. Furthermore, ion chromatography (IC) technique was used to measure the calcium ion concentration in the CaSO_4_ dissolved solution with different concentrations.

## Materials

Calcium Sulfate (CaSO_4_, anhydrous) was obtained from a supplier (Thermo Fisher Scientific) with purity of 99%. The converting agent, K_2_CO_3_ anhydrous was supplied by Thermo Fisher Scientific with purity above 99%. Tetrapotassium EDTA (C_10_H_12_K_4_N_2_O_8_) was supplied by Dayang chem (Hangzhou) company. The pH of the dissolver was adjusted using potassium hydroxide (KOH) which was purchased from Sigma Aldrich. The solutions were prepared using deionized (DI) water from MilliQ system.

## Experimental procedure

### Conversion process at high temperature

In this method, Calcium Sulfate (CaSO_4_) was converted to calcium carbonate (CaCO_3_) using a conversion agent, potassium carbonate (K_2_CO_3_), at high temperatures (200 °F) and under various high pH conditions (7, 10.5, and 12.5) resulting in potassium sulfate (K_2_SO_4_) which is considered as white water-soluble solid. The source of the high pH is potassium hydroxide (KOH). Potassium carbonate (K_2_CO_3_) was prioritized over sodium carbonate (Na_2_CO_3_) in this study due to its high thermal stability and solubility at high temperature. The chemical reaction is as follows:

1 $${\text{K4 - EDTA }} + {\text{K}}_{{2}} {\text{CO}}_{{3}} + {\text{ CaSO}}_{{4}} \mathop{\longrightarrow}\limits^{{{\text{high}}\,{\text{pH}}}}{\text{K}}_{{2}} {\text{SO}}_{{4}} + {\text{CaCO}}_{{3}} .$$

From reaction Eq. (1), it can be observed that EDTA and converting agent convert the CaSO_4_ into an acid or water-soluble product. The chelating agent complexes Ca ions from the scale and dissociated SO_4_ ions in the solution. The converting agent is water-soluble and dissociated into K ions and CO_3_ ions in the solution. K ions have a strong affinity for SO_4_ ions and make K_2_SO_4_, a water-soluble compound. Ca ions combine with CO_3_ ions and convert into CaCO_3_, an acid-soluble compound.

Tetrapotassium EDTA, a chelating agent, was used as a dissolver. The conversion and dissolution were carried out in a single step, subjecting the calcium sulfate to a dissolver containing K4-EDTA and the K_2_CO_3_.

To begin, K4-EDTA and K_2_CO_3_ were combined, and the pH was adjusted to the required value using KOH. The carboxyl groups of EDTA are not dissociated at low pH. The EDTA is usually dissolved by adding NaOH or KOH to EDTA. When the pH rises, more carboxyl become dissociated (COO–), releasing protons (H^+^) to the solution. Dissociated EDTA is ionic and thus water-soluble. Undissociated carboxyl (COOH) has no charge because the hydrogen is covalently bound, and therefore, acid EDTA is almost insoluble in water.

CaSO_4_ was added to the dissolver solution and incubated at a temperature of 200 °F for different time intervals (3, 6, 12 and 24 h). The experiment was carried out on a magnetic stirrer under continuous shearing of 300 rpm. At the end of the soaking period, the reaction product was filtered out and dried overnight at 176 °F. The dissolution efficiency was calculated using Eq. (2).

2 $${\text{Dissolution}}\,{\text{efficiency }} = { }\frac{Final\,weight\,after\,drying\,end\,product}{{Initial\,weight\,of\,CaSO_{4} }} \times 100.$$

**Figure 3:**
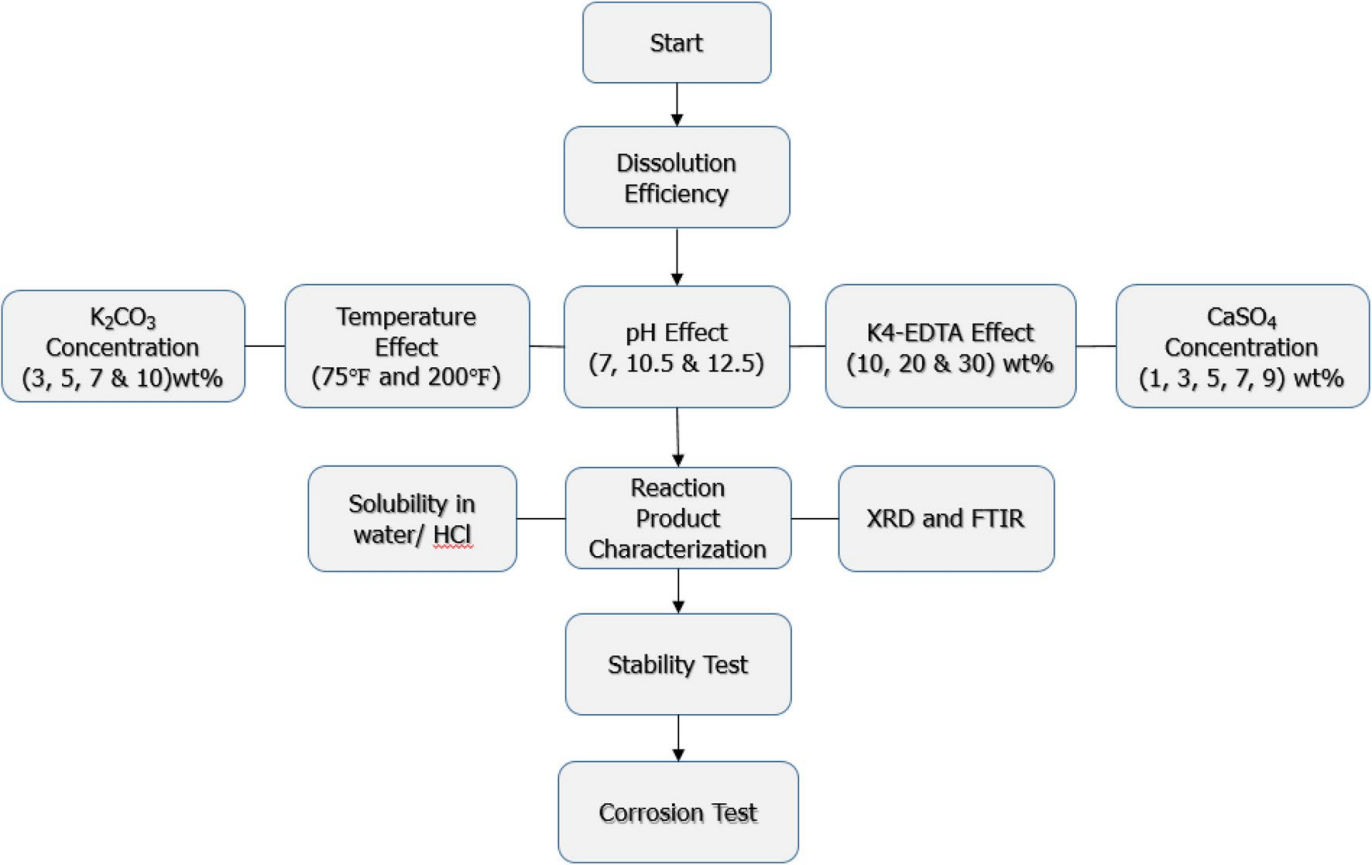
Flow chart of the single step scale removal study.

To evaluate the reaction product's solubility, it was dissolved in 15% HCl and DI water. The solubility of CaSO_4_ was investigated under various variables included pH, CaSO_4_ scale concentration, EDTA concentration, concentration of K_2_CO_3_, temperature and soaking time. Table 1 shows the testing parameters and values used in the study. In addition to solubility tests, the stability of the dissolvers and corrosion potential were investigated. Figure 3 provides flow chart of the study.

**Table 1:** Testing values used in the single step method to dissolve CaSO_4_ scale with K4-EDTA.

Experiment parameters	Values
pH of dissolvers	7, 10.5 and 12.5
CaSO_4_, wt%	1, 3, 5, 7 and 9
K_2_CO_3_ concentration in the mixed solution, wt%	5, 10 and 15
EDTA concentration, wt%	10, 20, and 30
Temperature, °F	75 and 200
Soaking time, hours	3, 6, 12 and 24

### FTIR and XRD characterization of reaction product

The reaction product's Fourier Transmission Infrared Spectroscopy (FTIR) was performed using FT-IR Spectrometer INVENIO from BRUKER by ATR technique. The FTIR technique is conducted to find out the functional group present in the reaction product. It was conducted from (4000–405) cm^−1^ wavelength range.

Furthermore, X-ray diffraction (XRD) analysis was performed to confirm the composition of the reaction product. The XRD analysis of the reaction solid product was studied using Powder XRD, PANanalytical Empyrean. The samples were scanned in the 2*θ* range of 0°–70° at a scan rate of 1°/s.

## Results and discussion

### Conversion process at high temperature

In this study, calcium sulfate scale was dissolved by a conversion process. The calcium sulfate was transformed into acid and chelating agent dissolvable product using a converting agent, potassium carbonate. A series of tests under various conditions were conducted to determine the optimal concentrations of K_2_CO_3_ and K4-EDTA for dissolving the CaSO_4_.

CaSO_4_ has very limited solubility in acids like HCl. It is commonly converted into acid soluble product using carbonate or bicarbonates. The converted product can be easily dissolved in HCl or other acids.

**Figure 4:**
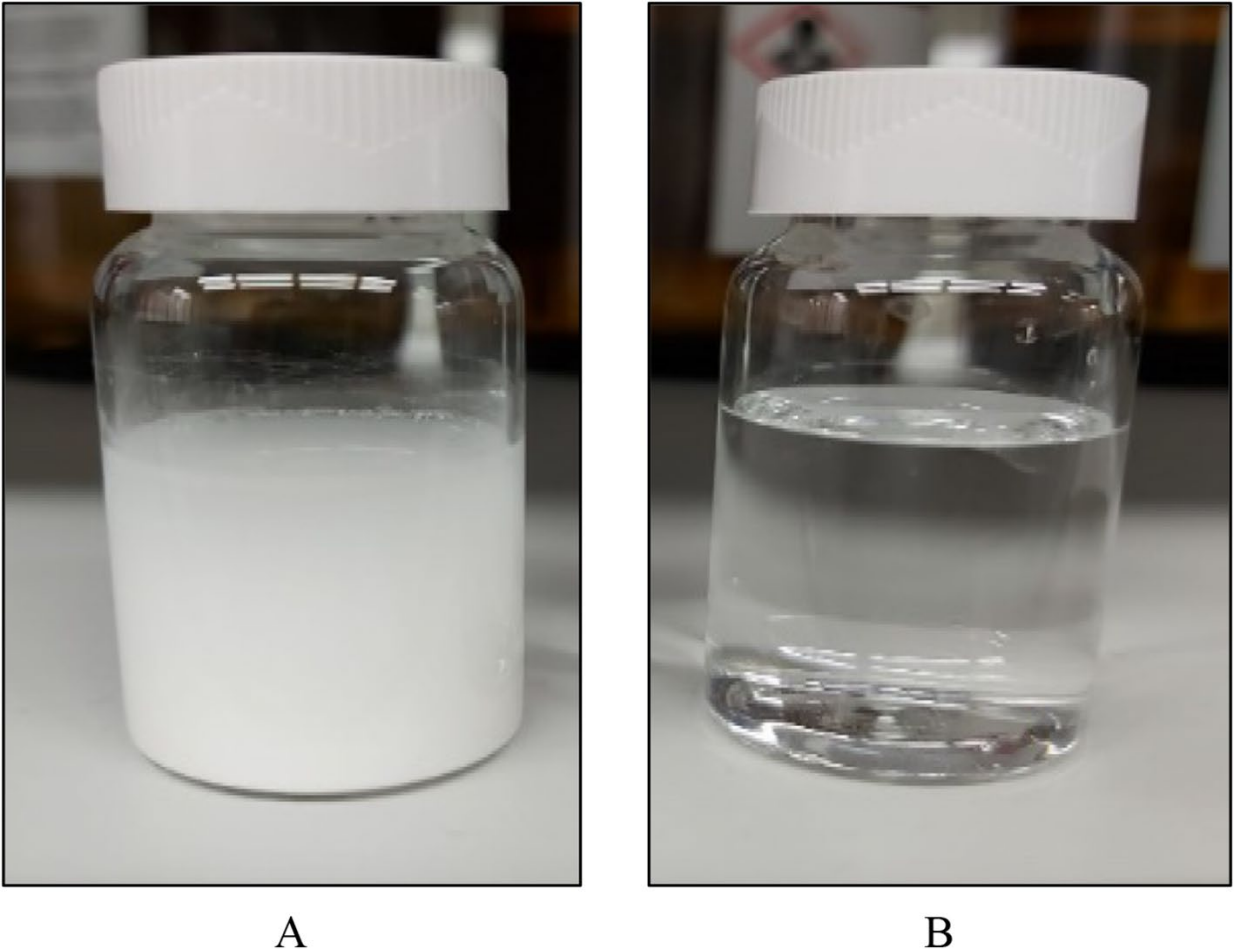
(**A**)The solution after reaction between CaSO_4_ and K_2_CO_3_, (**B**) The dissolution of the reaction product in 15 wt% HCl solution.

To confirm the conversion, 5 wt% CaSO_4_ was mixed with 5 wt% K_2_CO_3_ with a solution pH of 11.6 and soaked for 24 h at 200 °F. At the end of the soaking period, a milky colored solution was obtained (Fig. 4A). The reaction product was filtered and dissolved in a 15 wt% hydrochloric acid (HCl). The reaction product dissolved completely in HCl. It demonstrates that CaSO_4_ was converted during soaking in the presence of K_2_CO_3_ into CaCO_3,_ which is acid-soluble as shown in Fig. 4B.

**Figure 5:**
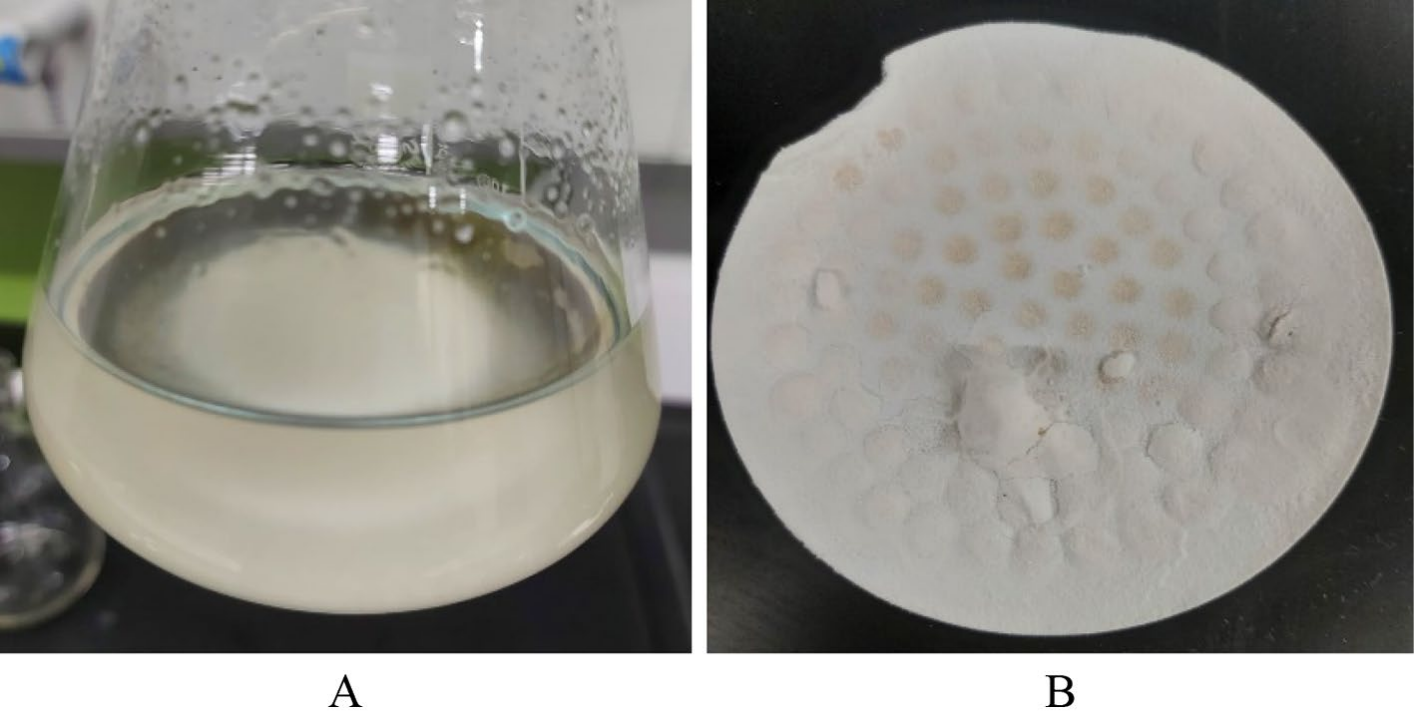
(**A**) Reaction product during soaking, (**B**) Reaction product after filtration (white powder).

Later, CaSO_4_ was mixed in dissolver solution prepared with K_2_CO_3_ and 20 wt% EDTA. The dissolution was evaluated under different variables, as shown in Table 1. CaSO_4_ was converted and dissolved by the dissolver. The reaction product was obtained in most of the tests conducted with high pH dissolvers (Fig. 5A). The reaction product was white in color and showed solubility in water and HCl, as shown in Fig. 5B.

The reaction product was analyzed by dissolving it in a solution of water and HCl. It was found to be soluble in both water and HCl. Additionally, it was observed that the reaction product increased the pH of the solution following dissolving, indicating that the final product was indeed K_2_SO_4_ produced as a result of the dissolver being excessively saturated with KOH.

### Effect of soaking time on dissolution

**Figure 6:**
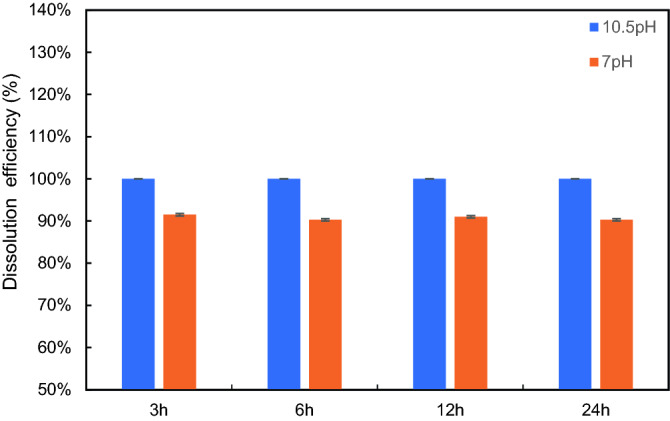
Effect of soaking time (3, 6, 12 and 24 h) on CaSO_4_ dissolution at 200 °F.

This study investigated the soaking time effect to obtain the optimum time for the maximum dissolution. The effect of soaking time was investigated by conducting dissolution of 5 wt% CaSO_4_ in two dissolvers that differ in pH (7 and 10.5) at 200 °F. The converting agent and EDTA concentrations were 5 wt% and 20 wt%, respectively. Figure 6 provides the results. It was noticed that the increase in soaking time did not improve dissolution—the maximum dissolution was achieved within three hours for a 10.5-pH dissolver. For the 7-pH dissolver, the efficiency did not improve and remained at 90–92% range. Based on this investigation, three hours was selected as a soaking time in most of the tests.

### The effect of dissolver pH

**Figure 7:**
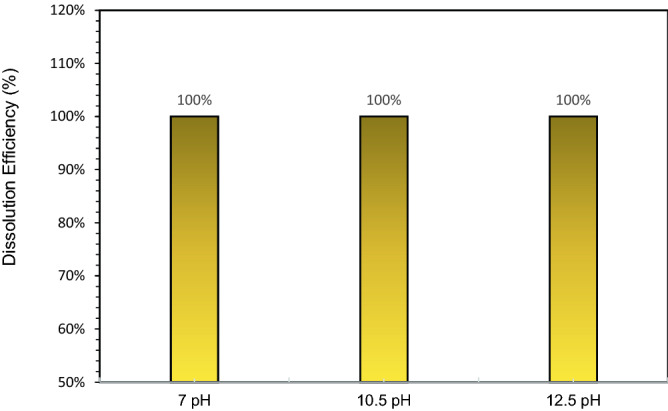
The effect of dissolver pH on 3 wt% CaSO_4_ dissolution.

**Figure 8:**
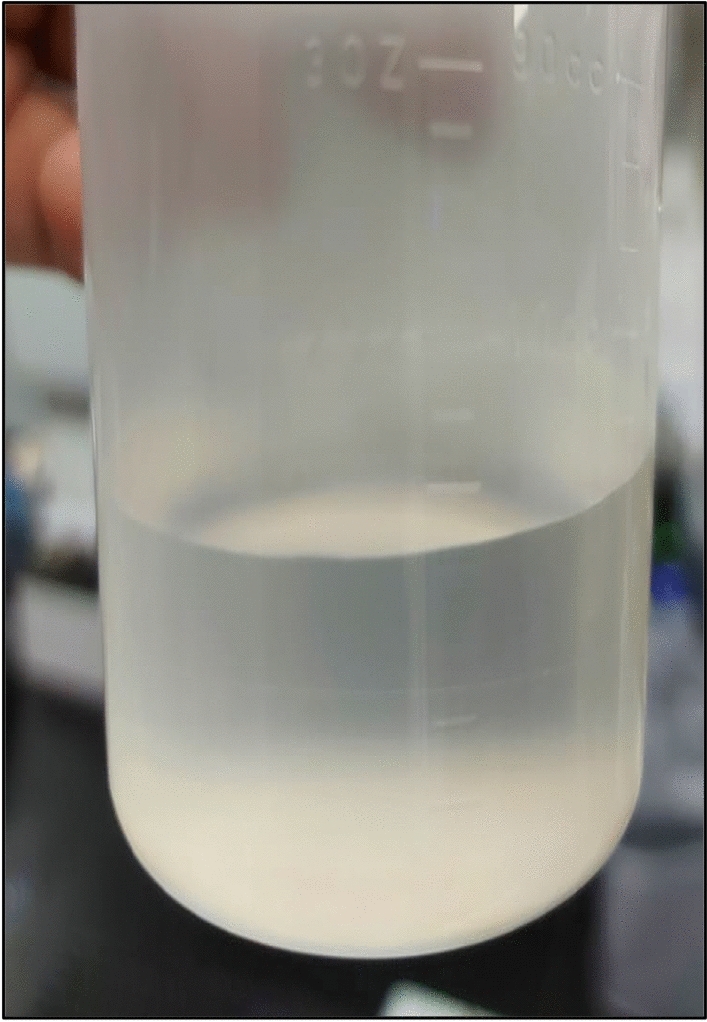
Final solution at the end of the test.

The dissolver pH has an impact on the dissolution of CaSO_4_. Figure 7 provides the dissolution efficiency with respect to change in pH. All tested dissolvers had the same formulation but differ in pH (7, 10.5 and 12.5). The dissolvers composed of 20 wt% K4-EDTA and 5 wt% K_2_CO_3_. The dissolution test was conducted on 3 wt% CaSO_4_ at 200 °C for 6 h as soaking time. It was observed that all the dissolvers dissolved the CaSO_4_ scale in soaking time at high temperature. After dissolution, the solutions were kept at room temperature for 24 h. There were precipitate in 10.5 and 12.5 dissolvers. On other hand, no precipitates were observed in the 7-pH dissolver (Fig. 8). With 7-pH dissolver, the combined effect of hydrogen ion assault and chelation accelerates dissolution. Calcium sulfate dissolving rate varies significantly with pH and chelating agent type due to variations in the chelating agent's ionic state and the impact of hydrogen ion assault^4^.

### Effect of CaSO_4_ concentration

**Figure 9:**
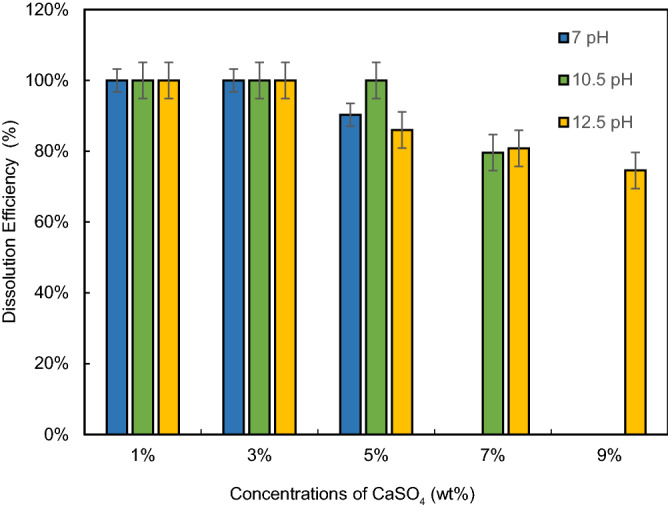
Dissolution efficiency vs. CaSO_4_ concentration at different pH dissolvers.

Figure 9 provides the dissolution efficiency of CaSO_4_ scale with respect to its concentrations dissolved in different pH solutions (7, 10.5 and 12.5). It was observed that the dissolution efficiency decreased with an increase in the concentration of CaSO_4_ in each dissolver solution. For instance, 7-pH dissolver provided 100% dissolution at 1 wt% and 3 wt% CaSO_4_ concentrations. At 5 wt%, the dissolution efficiency decreased to 90.3%. Similar behavior observed in other high pH dissolver solutions at different concentrations of CaSO_4_. For 10.5-pH dissolver, it showed better performance among other dissolvers. It dissolved CaSO_4_ completely up to 5 wt%. Further increase in concentration resulted in a reduction in efficiency due to excess CaSO_4_. At 5 wt%, it was observed that the 10.5-pH dissolver performed better than the 7-pH dissolver so the added concentration of CaSO_4_ are then tested against the 10.5- and 12.5-pH dissolvers. At 7 wt%, it was observed that the 12.5-pH dissolver performed better than the 10.5-pH dissolver and therefore the increased concentration (9 wt%) was tested against the 12.5-pH dissolver.

**Figure 10:**
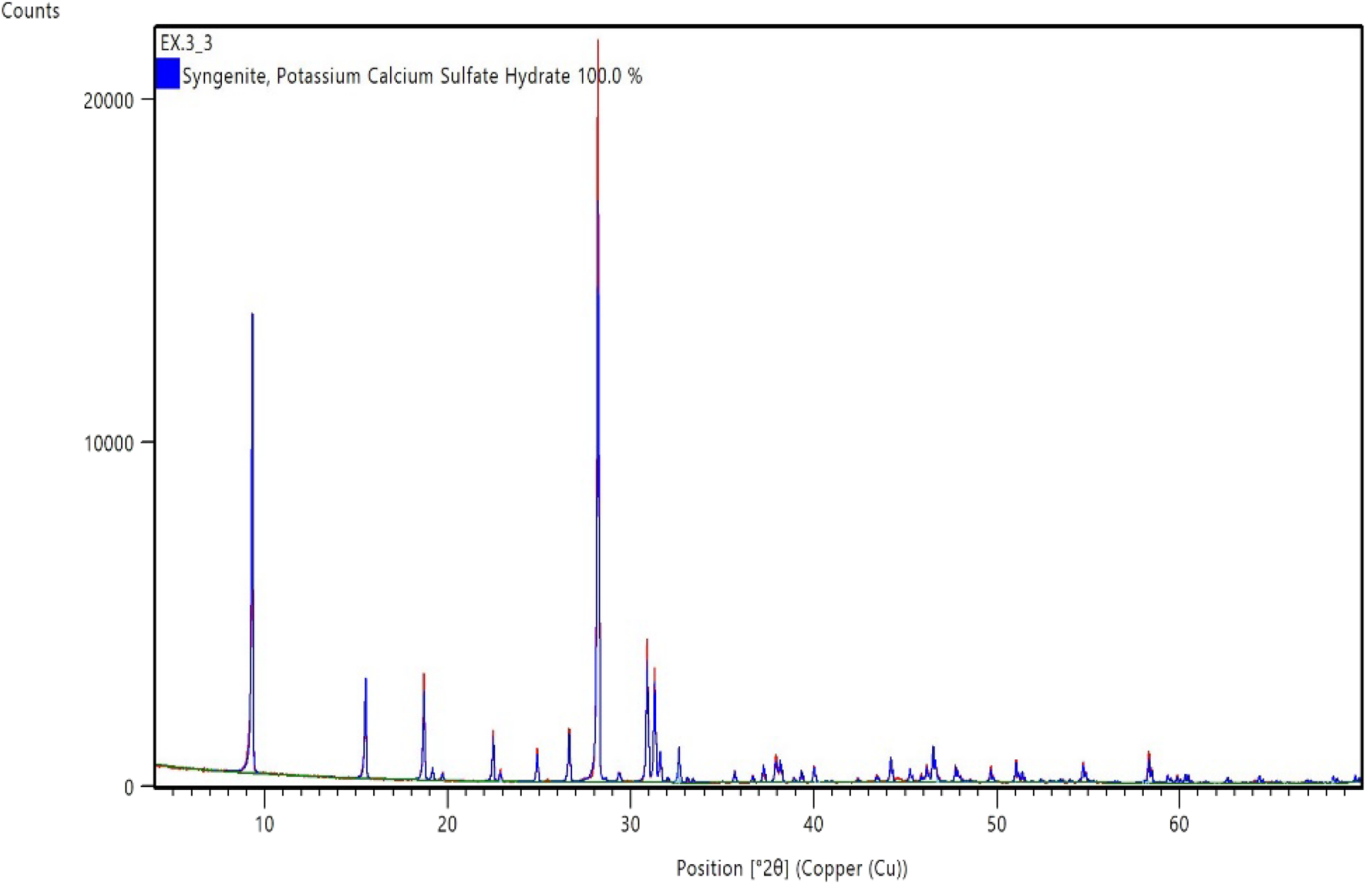
XRD spectrum of reaction product after the dissolution of 5 wt% CaSO_4_ in 7-pH dissolver and soaking at a room temperature condition.

**Figure 11:**
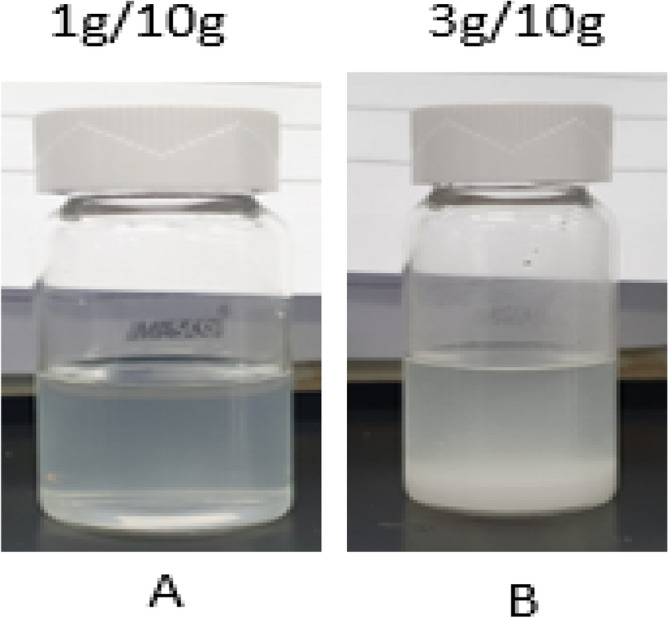
Solubility of the end product in water.

The reaction product obtained from dissolution of 5 wt% CaSO_4_ in 7-pH dissolvers was analyzed by XRD. Figure 10 shows the XRD spectrum of the reaction product. It was a mix of potassium calcium sulfate hydrate. From the XRD it was clear that the 7-pH dissolver could not convert the CaSO_4_ into acid or water-soluble product. Later, the end product solubility was investigated in water. It was noticed that addition of 1 g/100 ml end product in water produced a milky colored solution with some precipitates at the bottom of the vial (Fig. 11A). Further increase in concentration of reaction product to 3 g/100 ml provided more precipitations (Fig. 11B).

**Figure 12:**
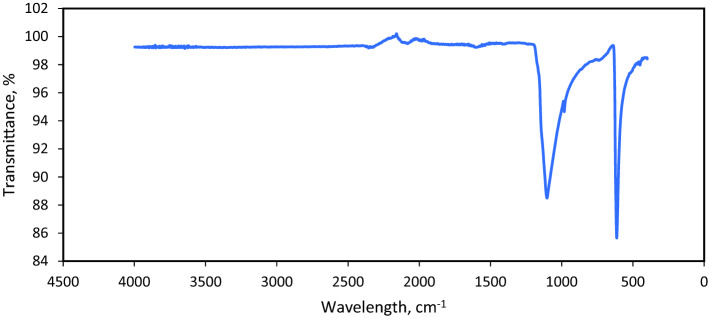
FTIR of the reaction product for 5 wt% CaSO_4_ dissolved in 10.5-pH dissolver.

Figure 12 shows the FTIR of the reaction product obtained from 5 wt% CaSO_4_ dissolution in 10.5-pH dissolver. The strong transmittance peaks were observed at 1100 cm^−1^, representing (C–O) stretch. The stretch at 613 cm^−1^ showed the C–X (halide) functional group. The measured FTIR of the reaction product agrees with FTIR in the literature^26^.

**Figure 13:**
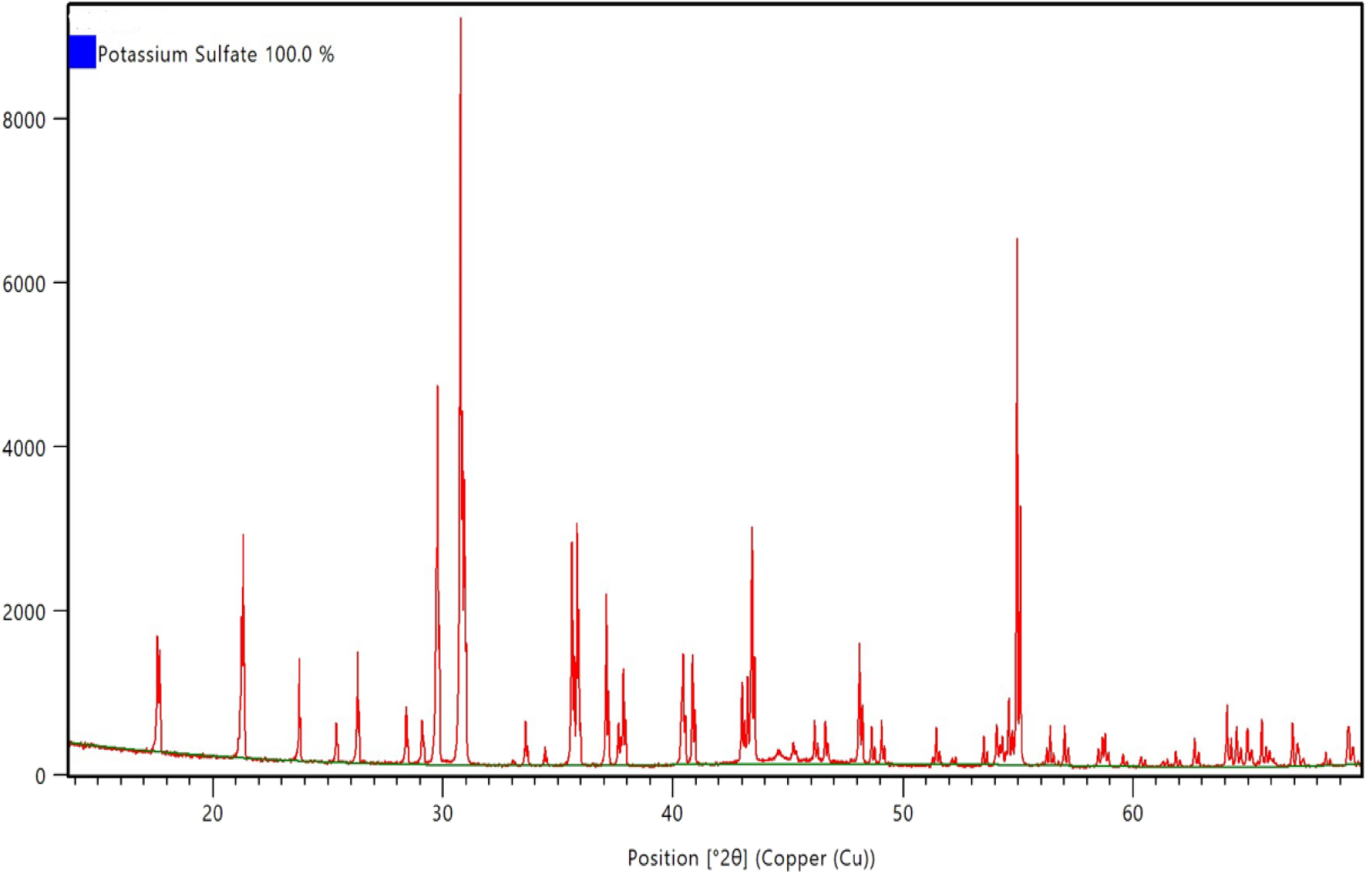
XRD spectrum of reaction product after the dissolution of 5 wt% CaSO_4_ in 10.5-pH dissolver.

Figure 13 shows the XRD pattern of the reaction product obtained at the end of the scale removal test. The XRD pattern is also in agreement with the literature^27^. From both analyses, it was found that the reaction product was K_2_SO_4_. The K_2_SO_4_ dissolves wholly and rapidly in water^28^. The K_2_SO_4_ can be removed by flushing with water after the soaking period.

**Figure 14:**
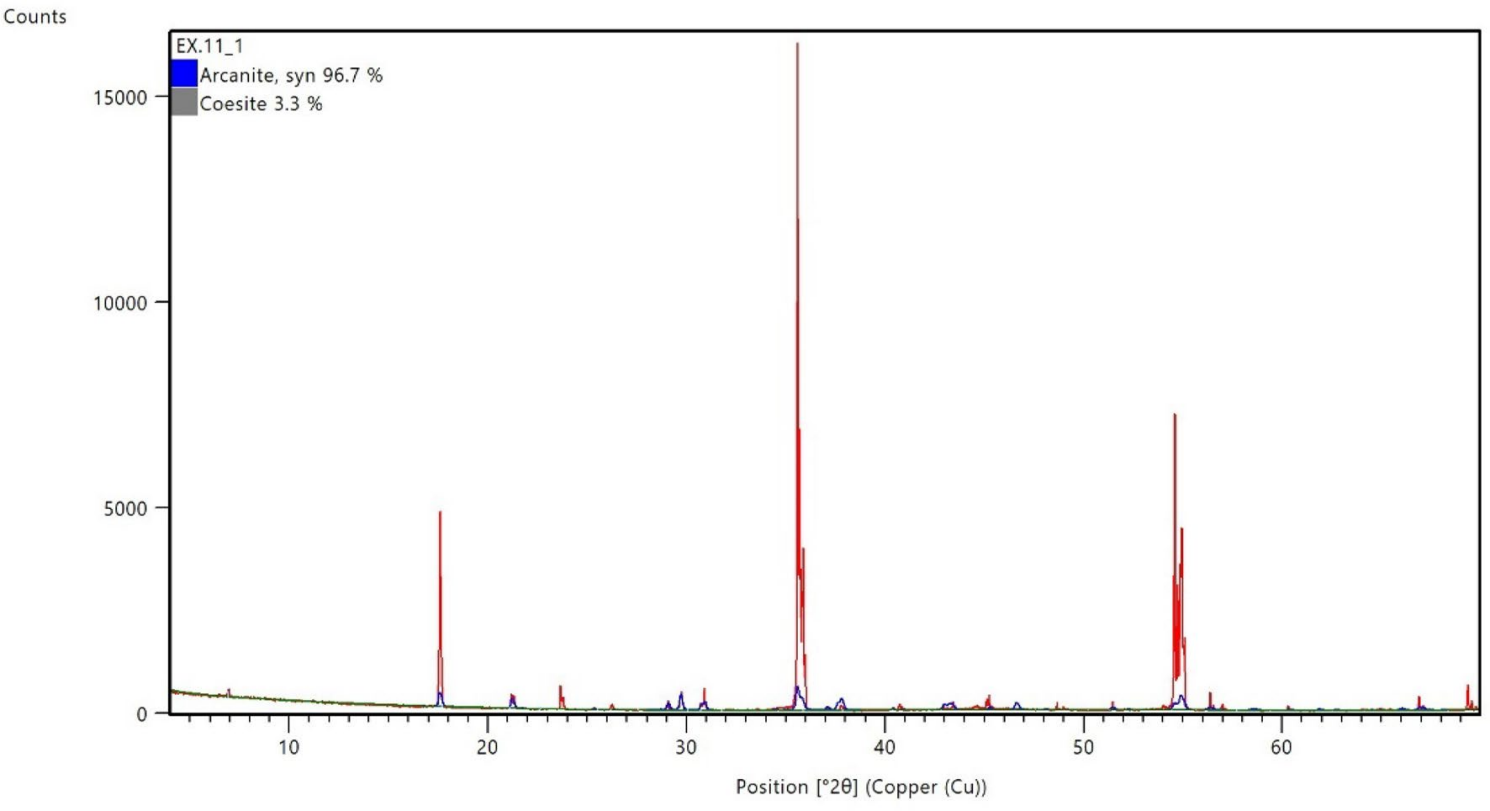
XRD of reaction product resulted from 7 wt% CaSO_4_ dissolution in 10.5-pH dissolver.

**Figure 15:**
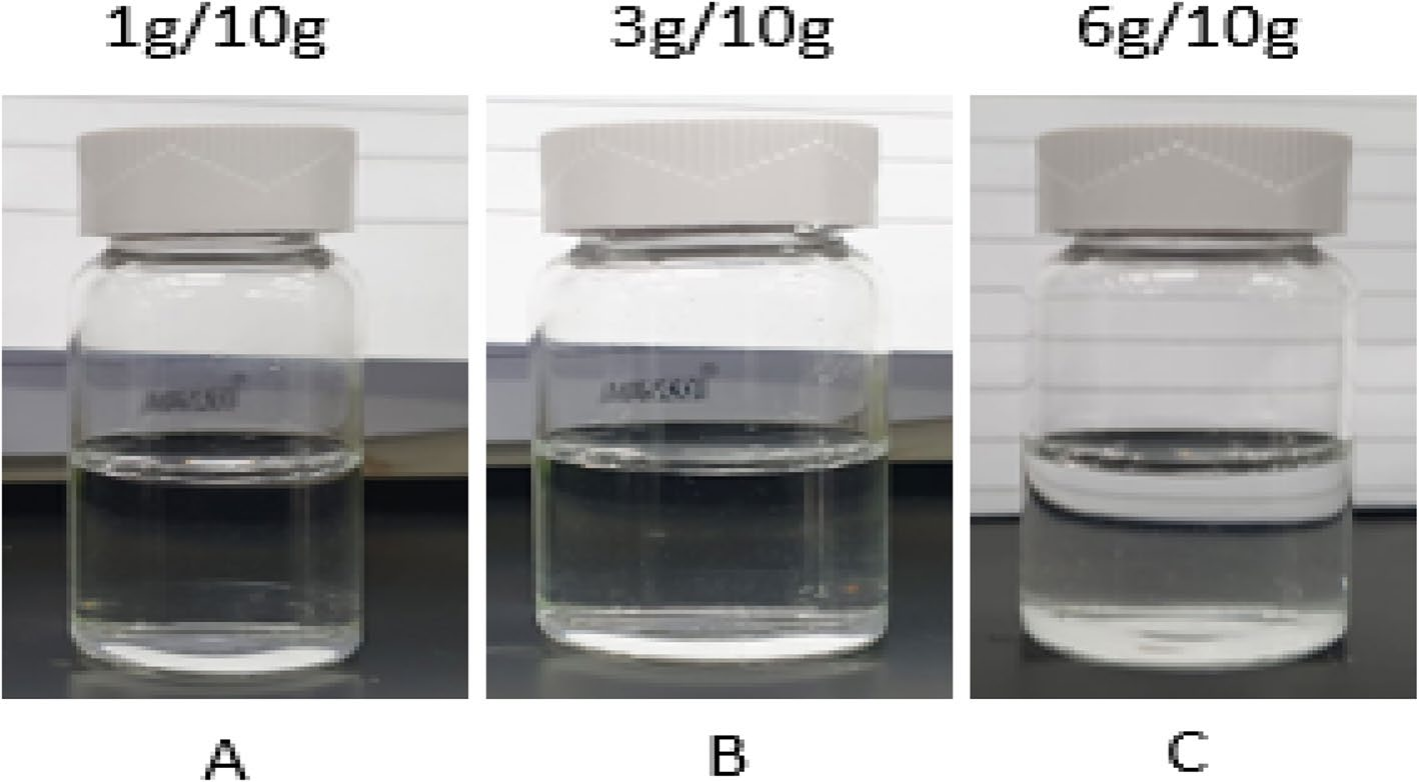
Solubility of reaction product in water after the dissolution of 7 wt% CaSO_4_ in 10.5-pH dissolver.

The 10.5-pH dissolver able to convert the 7 wt% CaSO_4_ into water soluble product. The end product was evaluated from XRD and potassium sulfate (arcanite) was diagnosed as the main reaction product (Fig. 14). The reaction product was completely dissolved in water as shown in Fig. 15.

**Figure 16:**
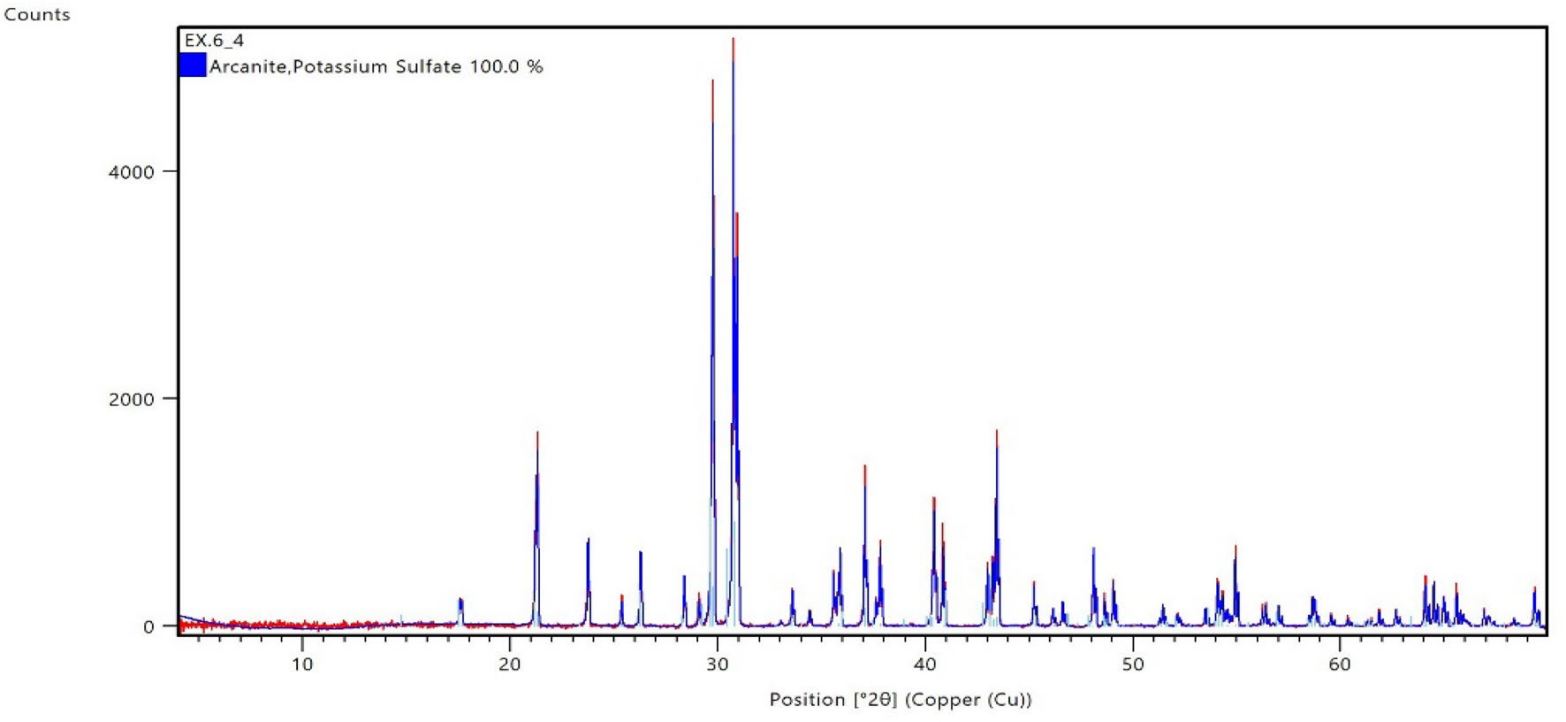
XRD spectrum of reaction product after the dissolution of 5 wt% CaSO_4_ in 12.5-pH dissolver at 200 °F.

The 12.5-pH dissolver showed low efficiency for 5 wt% and onward concentrations of CaSO_4_ as shown in Fig. 7. There was an appreciable reduction in dissolution efficiency at 5 wt% and onward. It resulted in high concentration of reaction product at the end of soaking period. The reaction product was analytically analyzed using XRD technique and found out potassium sulfate as the main product in 5 wt% CaSO_4_ dissolution as shown in Fig. 16.

**Figure 17:**
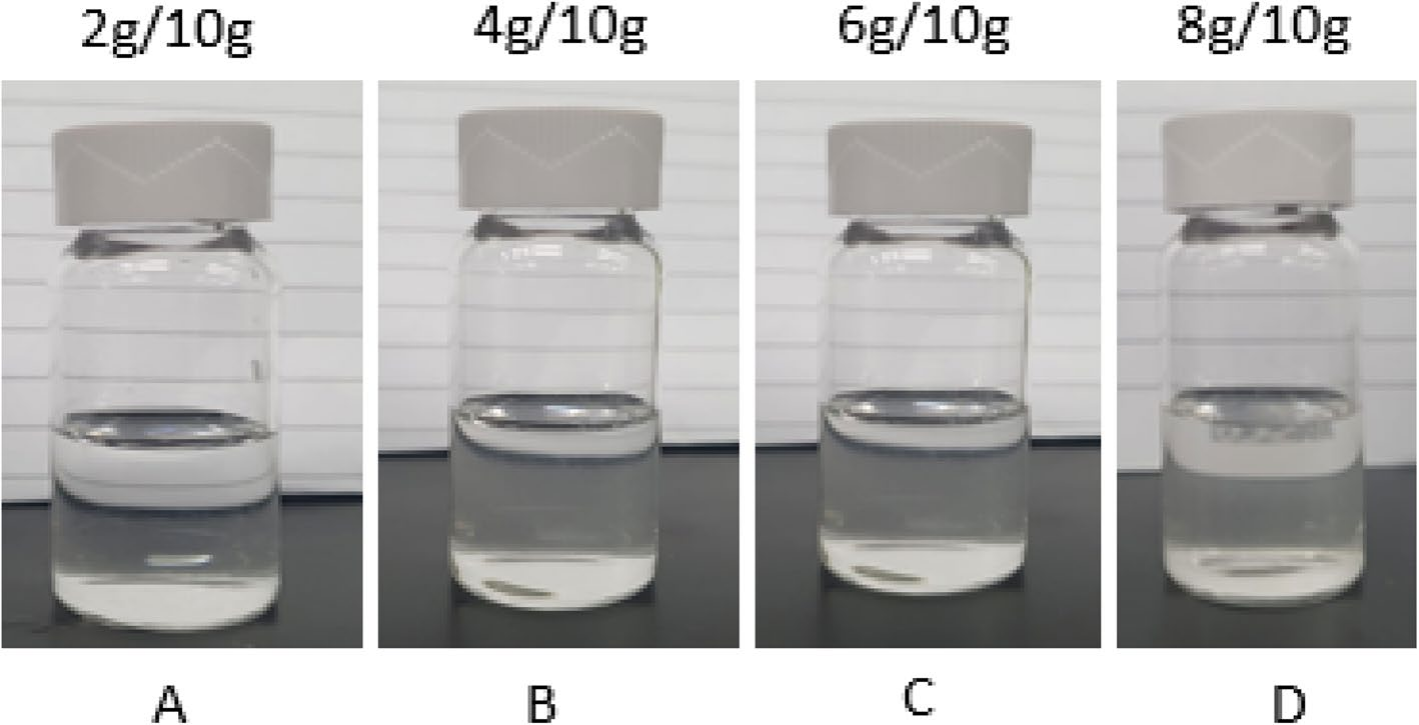
Solubility of the reaction product in water.

**Figure 18:**
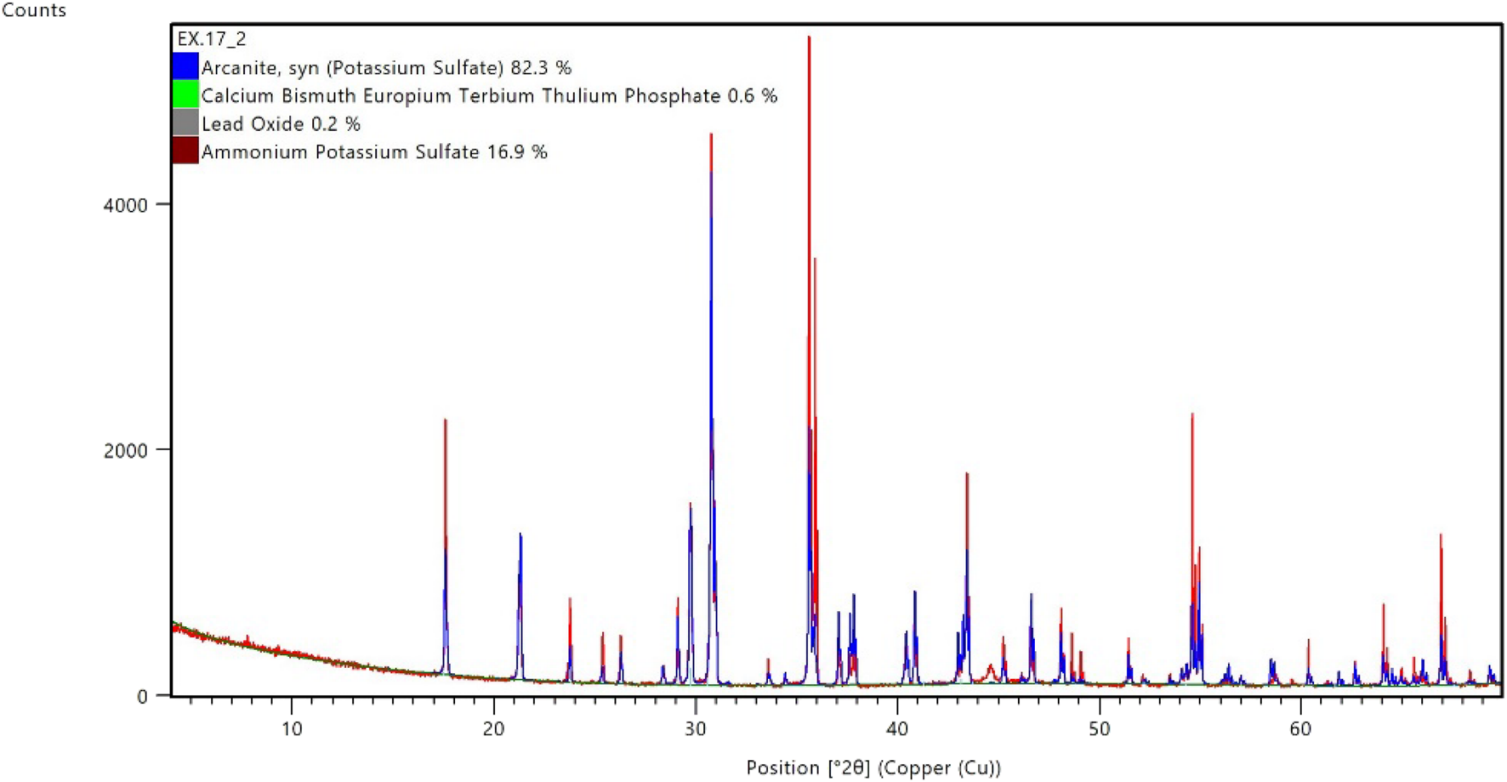
XRD spectrum of reaction product after the dissolution of 9 wt% CaSO_4_ in 12.5-pH dissolver at 200 °F.

The reaction product was dissolved in water at different concentration. It was completely dissolved in water up to 6 g/100 ml (Fig. 17A–C). At 8 g/100 ml concentration, the reaction product reached saturation and precipitated at the bottom of solution (Fig. 17D). The reaction resulted at 9 wt% CaSO_4_ showed incomplete dissolutions and resulted in different compounds at the end of soaking period as shown in Fig. 18.

**Figure 19:**
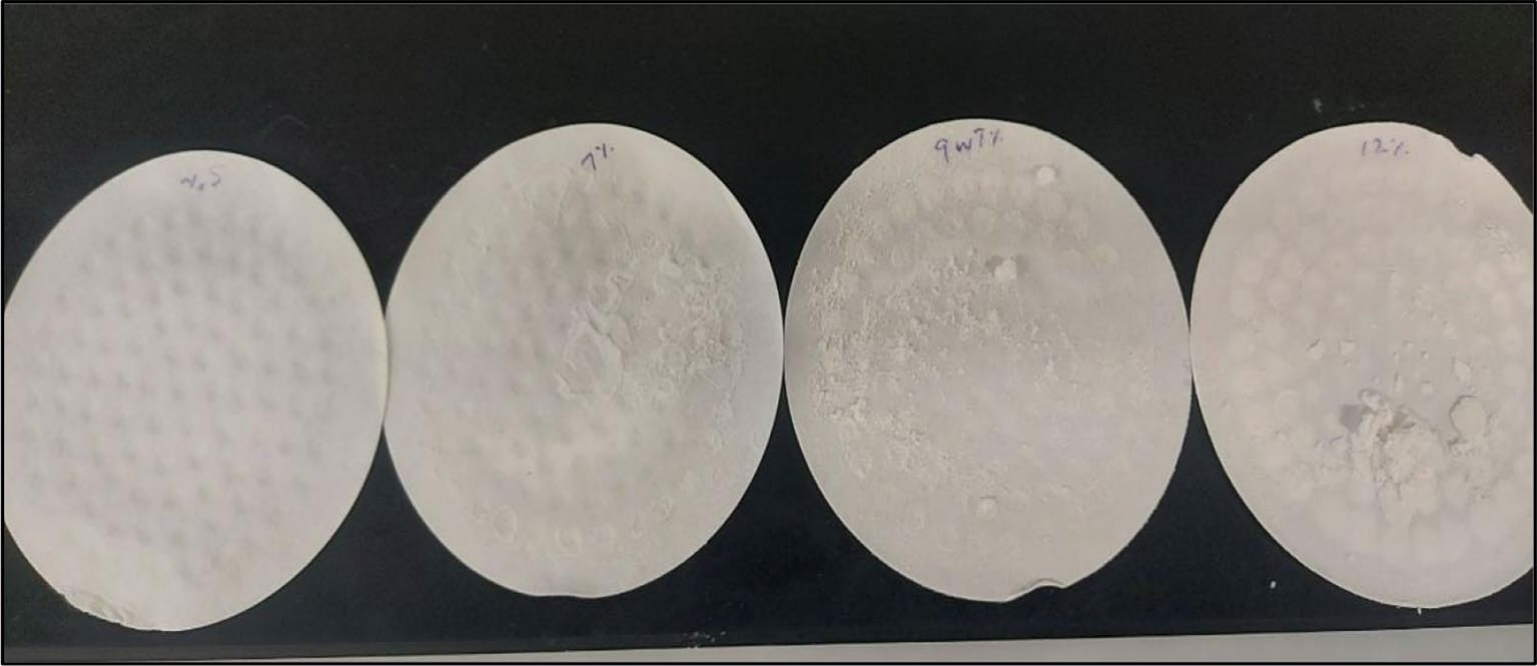
Reaction products after dissolution of CaSO_4_ at different concentrations.

To investigate the dissolution efficiency of 10.5-pH dissolver, various concentrations of CaSO_4_ (3,5, 7, 9 and 12) wt% were dissolved and kept at 200 °F for 6 h. The reaction products were filtered out at the end of the soaking time, as shown in Fig. 19. It was observed that there is a concentration effect on the dissolution efficiency. The amount of reaction product increases with the rise in the concentration of CaSO_4_, showing the reduction in dissolution efficiency.

**Figure 20:**
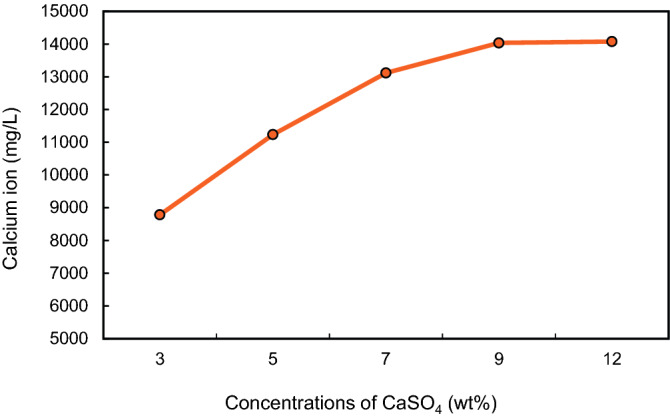
Calcium ions concentrations in CaSO_4_ dissolved solutions (3, 5, 7, 9 and 12) wt%.

Further, the calcium ions concentrations were measured by applying the ion chromatography (IC) technique. It was observed that calcium ions concentrations increased with an increase in CaSO_4_ concentration. The rising trend was noticed up to 9 wt%. At 12 wt%, there was little change in Calcium ions dissolution, the curve reached its plateau. The dissolved calcium ions concentrations were 13,117 mg/l and 14,034 mg/l at 9 wt% and 12 wt%, respectively (Fig. 20).

### Effect of EDTA concentration

**Figure 21:**
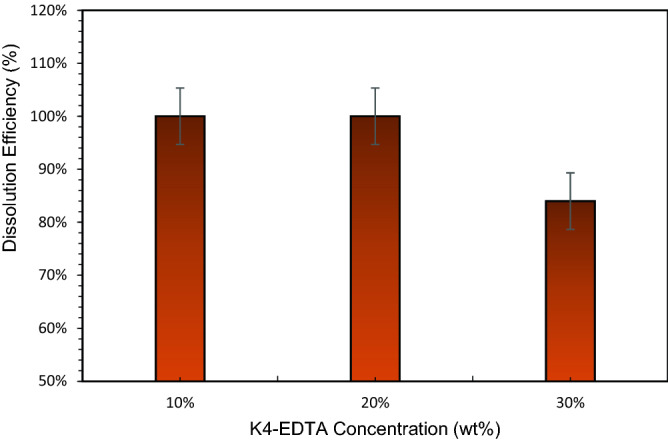
Dissolution efficiency vs. K4-EDTA concentration.

Figure 21 shows the effect of K4-EDTA concentration on CaSO_4_ dissolution. In this study, the CaSO_4_ concentration was maintained at 5 wt%. The test was conducted at 200 °F for 6 h using 10.5-pH dissolver. It was observed that the dissolution efficiency was maintained at 100% for 10 wt% and 20 wt% K4-EDTA concentrations. Further increase in concentration to 30 wt% reduced the dissolution efficiency to 84%. At high EDTA concentration, solids remained at the end of the 3 h soaking period.

### Effect of different concentrations of K_2_CO_3_

**Figure 22:**
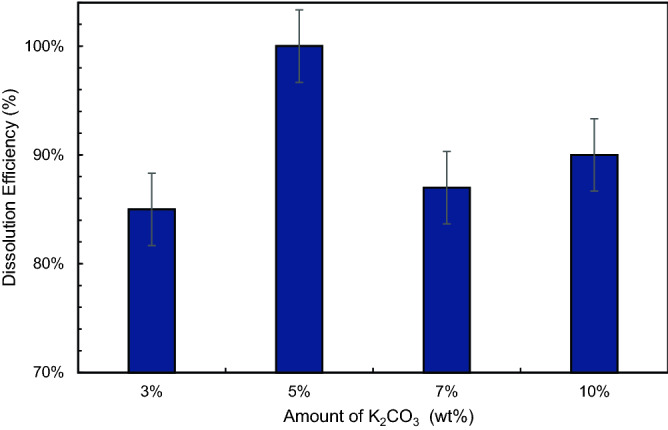
Dissolution efficiency vs. concentration of K_2_CO_3_.

The convertor concentration has an impact on the solubility of scale. Figure 22 shows the concentration effect of K_2_CO_3_ on CaSO_4_ dissolution. In this part, the CaSO_4_ concentration was maintained at 5 wt%. The test was conducted at 200 °F using 10.5-pH dissolver. The EDTA concentration was 20 wt% in all the solutions. Only K_2_CO_3_ concentration was varied from 3 to 10 wt%. It was observed that K_2_CO_3_ impacted the dissolution efficiency. The dissolution efficiency varied with a change in concentration. The optimum dissolution was observed at 5 wt% of K_2_CO_3_. Further increase in concentration did not change the efficiency appreciably showed ineffectiveness at high concentration. The dissolution efficiency at 7 wt% and 10 wt% were 87% and 90%, respectively.

### Effect of temperature

**Figure 23:**
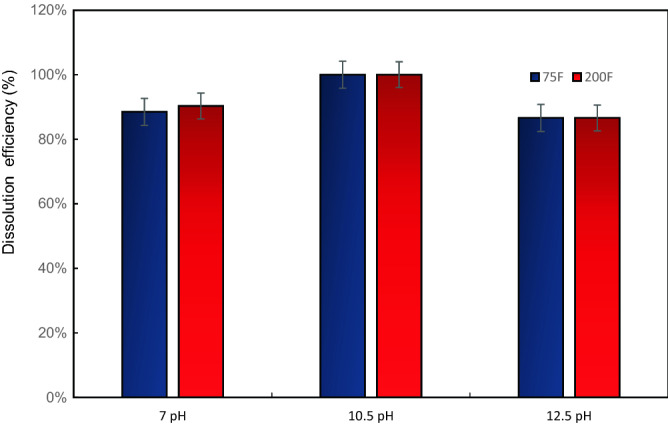
Effect of temperature on dissolution efficiency for different pH dissolver solutions.

The temperature has a minor impact on CaSO_4_ dissolution efficiency for tested dissolvers, as shown in Fig. 23. The study was conducted at two different temperatures (75 °F and 200 °F). There was a negligible impact of temperature on dissolution observed. For instance, the dissolution efficiency slightly increased to 90.3% from 88.50% for 7-pH dissolver after temperature increased from 75 to 200 °F. For 10.5-pH, the dissolution efficiency was 100% at 75 °F and 200 °F. For 12.5-pH, there was no change observed in dissolution efficiency.

For 10.5-pH dissolver, it dissolved CaSO_4_ scale at low temperature and high temperature. In high temperature dissolution, when the solution temperature brought to room temperature, it resulted in precipitations. For low temperature, there was no precipitations observed.

In addition, the test of was conducted at static condition without stirring. There was no appreciable difference observed between stirring and non-stirring samples. 3 wt% CaSO_4_ was completely dissolved in stirring and non-stirring scenarios.

Further, the dissolution was investigated in freshwater from a tap. The 10.5-pH dissolver was mixed in tap water instead of DI water. Tap water has high concentrations of Ca and Mg ions compared to DI water. The 5 wt% CaSO_4_ was dissolved and compared its performance with dissolver prepared using DI water. It was observed that dissolver prepared with tap water resulted in 100% dissolution efficiency at 200 °F same as the dissolver prepared with DI water. After the dissolution, the solution was kept at room temperature and precipitation was occurred too in tap water mixed dissolver.

### Stability test

The stability test was conducted to investigate any physical or chemical change in the dissolver solution over time under high temperature conditions. In this test, two dissolver products that differ in pH were placed under a high temperature of 248 °F for three days.

**Figure 24:**
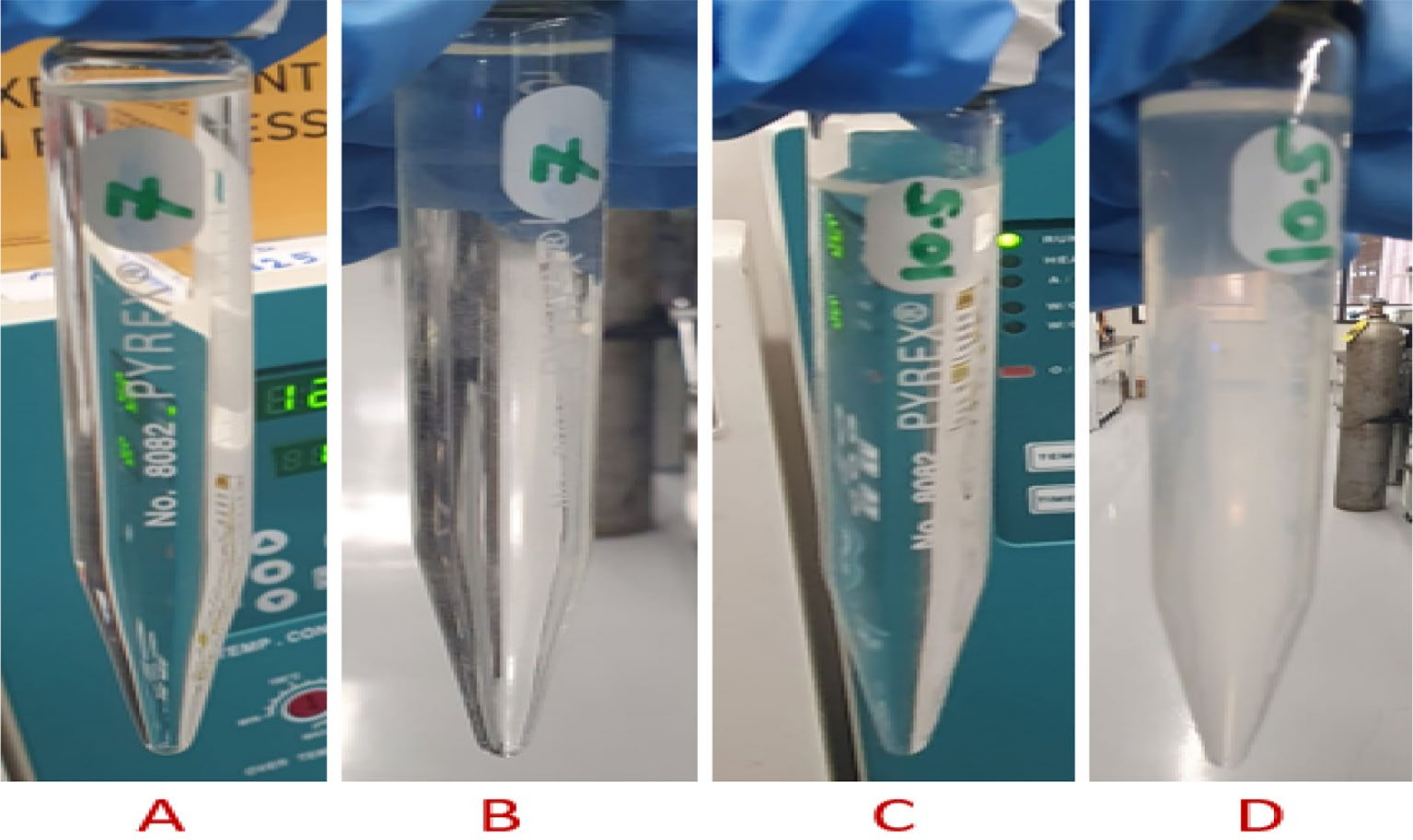
Stability tests of dissolvers at 248 °F.

It was noticed that there was no physical change in the solutions apparently before and after the exposure at high temperature, as shown in Fig. 24A, C. The solutions did not show any precipitation and color change after 3 days. Further, an interesting observation was made regarding the dissolver of 10.5-pH when it was placed under room conditions for 24 h after exposure to high temperature. The solution color changed to cloudy with a precipitate at the bottom of the test tube (Fig. 24D). The 7-pH dissolver solution did not result in any precipitate after keeping it at room temperature, as shown in Fig. 24B.

### Corrosion test

Corrosion testing is typically used to determine the corrosiveness of acids used in the oil and gas industry to ensure that they will not corrode the tubing and downhole completion tools due to their high corrosion rate. Additionally, it ensures that the acid does not cause damage to the equipment used to mix and inject chemicals into wells or surface flowlines. The test determines the fluid's effect on a steel coupon made of coiled tubing material by calculating the corrosion rate after soaking the steel coupons in the fluids at 149 °F temperature for 24 h using an OFITE corrosion cell. Corrosion tests were conducted on steel coupons (1/16″ × ¾″ × 3″) were conducted using DI water and two dissolver solutions. The dissolver solutions have identical formulations but differ in pH values (7 and 10.5). The controlled test was carried out using DI water as a reference.

**Figure 25:**
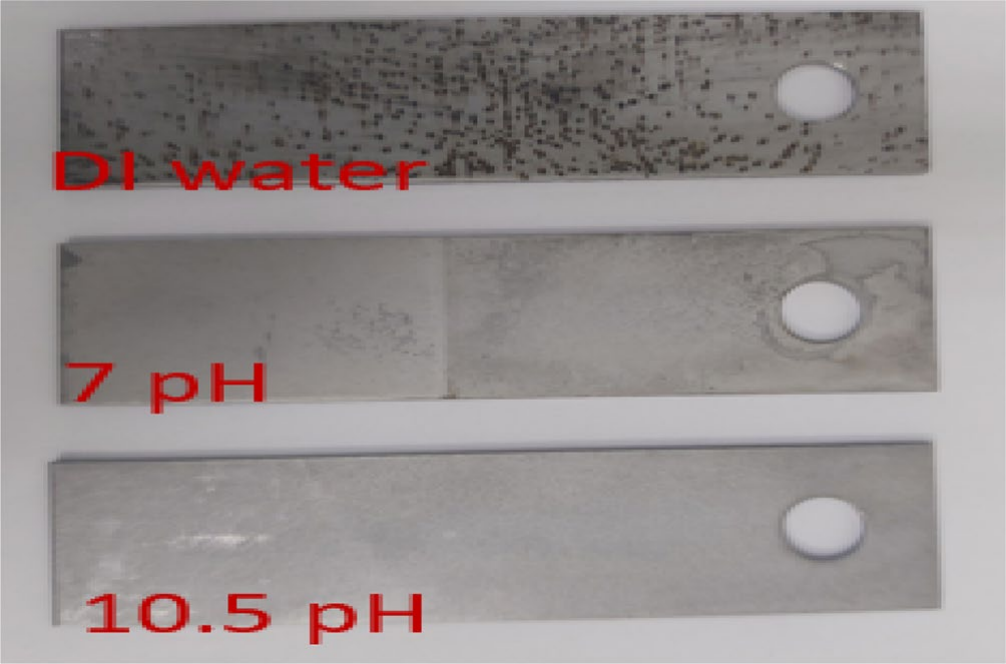
Corrosion coupons after soaking in solutions for 24 h.

It was observed that DI water resulted in high corrosion of 3.73 lbs/ft^2^/year compared to dissolver solutions, as shown in Table 2. The coupon soaked in water showed clear corroded marks and its weight was reduced (Fig. 25). The corrosion caused by water was higher than the recommended maximum limit value of 2 lbs/ft^2^/year. The deionized water is lacking ions and hungrier for the ions. The pH of the deionized water sometimes found to be slightly acidic. The low pH water cause corrosion in carbon steel upon contact. This could be the reason of high corrosion rate observed in DI water. Further, it was noticed that both dissolvers resulted in a low corrosion rate and corrosion was in the safe limit (< 2 lbs/ft^2^/year). The dissolver of 10.5-pH provided better corrosion protection as it caused the lowest corrosion rate (0.58 lbs/ft^2^/year) compared to other solutions.

**Table 2:** Corrosion rate of DI water, 7-pH dissolver and 10.5-pH dissolver on steel coupons.

Fluids	Formulations	Initial weight, g	Final weight, g	Corrosion rate, lbs/ft^2^/year
DI water	Water	15.09	14.94	3.73
7-pH dissolver	20 wt% K4-EDTA + 5 wt% K_2_CO_3_ + KOH as required	15.00	14.94	1.38
10.5-pH dissolver	20 wt% K4-EDTA + 5 wt% K_2_CO_3_ + KOH as required	15.24	15.21	0.58

## Conclusion

In this study, the removal of calcium sulfate scale has been investigated using a single step method utilizing potassium carbonate and K4-EDTA at high temperature. Various parameters were investigated to obtain a dissolver composition at which optimum dissolution efficiency obtained including the effect of dissolver pH, soaking time, the concentration of K4-EDTA, the concentration of K_2_CO_3_, the concentration of CaSO_4_, temperature impact, and agitation effect. The following are the conclusions that were drawn from this study:

1. The single step method which formed a dissolver solution containing K_2_CO_3_ and K4-EDTA, efficiently dissolved the CaSO_4_ scale at high temperature conditions (200 °F) by forming a reaction product (K_2_SO_4_) that is soluble in both water and HCl.
2. Precipitation of water or acid soluble product occurred in most tests performed at high pH.
3. There was a noticeable effect of pH on CaSO_4_ dissolvable concentration. Up to 3 wt% (30 g/l) of CaSO_4_ was entirely dissolved in 7-pH dissolver while 5 wt% (50 g/l) was only dissolved in 10.5-pH dissolver without precipitation or crystallization at 200 °F temperature.
4. The lowest corrosion rate was observed in both 7-pH and 10.5-pH dissolvers compared to water.
5. Both dissolvers were effective and showed high stability at 248 °F temperature.
6. The end product of the single step conversion process has been characterized and showed it has no harmful effects and can be dissolved rapidly and completely in water.
7. The single step dissolution process showed effectiveness and could potentially save significant pumping time if implemented in operation.

## Data Availability

The datasets used and/or analyzed during the current study available from the corresponding author on reasonable request.
